# Non-pharmaceutical public health interventions for pandemic influenza: an evaluation of the evidence base

**DOI:** 10.1186/1471-2458-7-208

**Published:** 2007-08-15

**Authors:** Julia E Aledort, Nicole Lurie, Jeffrey Wasserman, Samuel A Bozzette

**Affiliations:** 1 RAND Center for Domestic and International Health Security, 1776 Main Street, Santa Monica, California, USA; 2 University of California San Diego, San Diego, California, USA

## Abstract

**Background:**

In an influenza pandemic, the benefit of vaccines and antiviral medications will be constrained by limitations on supplies and effectiveness. Non-pharmaceutical public health interventions will therefore be vital in curtailing disease spread. However, the most comprehensive assessments of the literature to date recognize the generally poor quality of evidence on which to base non-pharmaceutical pandemic planning decisions. In light of the need to prepare for a possible pandemic despite concerns about the poor quality of the literature, combining available evidence with expert opinion about the relative merits of non-pharmaceutical interventions for pandemic influenza may lead to a more informed and widely accepted set of recommendations. We evaluated the evidence base for non-pharmaceutical public health interventions. Then, based on the collective evidence, we identified a set of recommendations for and against interventions that are specific to both the setting in which an intervention may be used and the pandemic phase, and which can be used by policymakers to prepare for a pandemic until scientific evidence can definitively respond to planners' needs.

**Methods:**

Building on reviews of past pandemics and recent historical inquiries, we evaluated the relative merits of non-pharmaceutical interventions by combining available evidence from the literature with qualitative and quantitative expert opinion. Specifically, we reviewed the recent scientific literature regarding the prevention of human-to-human transmission of pandemic influenza, convened a meeting of experts from multiple disciplines, and elicited expert recommendation about the use of non-pharmaceutical public health interventions in a variety of settings (healthcare facilities; community-based institutions; private households) and pandemic phases (no pandemic; no US pandemic; early localized US pandemic; advanced US pandemic).

**Results:**

The literature contained a dearth of evidence on the efficacy or effectiveness of most non-pharmaceutical interventions for influenza. In an effort to inform decision-making in the absence of strong scientific evidence, the experts ultimately endorsed hand hygiene and respiratory etiquette, surveillance and case reporting, and rapid viral diagnosis in all settings and during all pandemic phases. They also encouraged patient and provider use of masks and other personal protective equipment as well as voluntary self-isolation of patients during all pandemic phases. Other non-pharmaceutical interventions including mask-use and other personal protective equipment for the general public, school and workplace closures early in an epidemic, and mandatory travel restrictions were rejected as likely to be ineffective, infeasible, or unacceptable to the public.

**Conclusion:**

The demand for scientific evidence on non-pharmaceutical public health interventions for influenza is pervasive, and present policy recommendations must rely heavily on expert judgment. In the absence of a definitive science base, our assessment of the evidence identified areas for further investigation as well as non-pharmaceutical public health interventions that experts believe are likely to be beneficial, feasible and widely acceptable in an influenza pandemic.

## Background

Ongoing concerns about the emergence of an influenza pandemic continue as the number of avian and human infections with the H5N1 virus mount [1,2]. Adequate amounts of vaccine or antivirals are unlikely to be available early on in a pandemic, and the latter could become ineffective because of resistance [3]. These factors have focused attention on the use of non-pharmaceutical public health interventions to inhibit human-to-human transmission and fueled interest in answering important questions about influenza epidemiology and transmission [4-9]. However, the most comprehensive assessments of the literature to date recognize the generally poor quality of evidence on which to base pandemic planning decisions [8-10]. In light of the need to prepare for a possible pandemic despite concerns about the poor quality of the literature, combining available evidence with expert opinion about the relative merits of non-pharmaceutical interventions for pandemic influenza may lead to a more informed and widely accepted set of recommendations.

At the request of the US. Department of Health and Human Services, we evaluated the evidence base for non-pharmaceutical public health interventions by reviewing recent published literature, including historical reviews, convening a meeting of experts, and formally eliciting and quantifying expert opinion about the relative efficacy and effectiveness of specific non-pharmaceutical interventions for pandemic influenza. Based on this collective evidence, we identified a set of recommendations for and against interventions that are specific to both the setting in which an intervention may be used and the pandemic phase, and which can be used by policymakers to prepare for a pandemic until scientific evidence can definitively respond to planners' needs. We also identified important areas of uncertainty that warrant further research.

## Methods

### Literature review

We identified scientific articles through a MEDLINE search that combined communicable and infectious disease with non-vaccine and non-pharmaceutical intervention search terms. We searched various combinations of the Medical Subject Headings (MeSH) headings in biomedical and infections control journals back to 1966 [see Additional file 1]. We also performed additional searches for review articles in the following journals from 2000 to 2005: *Nature*, *Journal of the American Medical Association*, *New England Journal of Medicine*, *Annals of Internal Medicine*, *British Medical Journal*, *Science*, *Lancet*, *Emerging Infectious Diseases*, *Journal of Infectious Disease*, and *Clinical Infectious Disease*. Finally, we included additional literature identified by manual review of references lists of selected articles. The search identified 2,552 titles, of which 168 were eventually selected based on general relevance. Exclusion criteria are presented in Table 1. Using a modified rating scale derived from West et al., we then formally rated the strength of the scientific evidence presented in each of the relevant articles [11]. Table 2 presents the modified rating scheme and reports the number of individual articles that fell into each rating category. We have presented a list of the final articles selected for full review and classification [see Additional file 2].

**Table 1:** Results of literature search

Titles identified	2,556
Remaining after exclusion of case reports and articles pertaining to vaccines, antiviral medications other medical countermeasures	506
Remaining after title and abstract review	319
Remaining after screen for relevance	168

**Table 2:** Modified rating scheme and results for N = 168 articles

**Classification**	**Definition**	**Grade**	**Results**
Systematic Review	Documented extensive literature search; quantitative or qualitative summary	A	9
Narrative Review	Summary of field or problem by an expert citing references obtained in a non-systematic manner	B	49
RCT	Prospectively randomized at individual or group level	C	3
Observational Studies	Formal design, almost always a control group whether prospective or retrospective, and whether cross-section, panel, or case-control. Includes analysis of large dataset analysis.	D	29
Mathematical Models	Uses mathematical language to describe and predict biomedical and epidemiologic outcomes.	E	12
Case Reports or Series	Report of a (hopefully pertinent) case or series of cases, often with a perspective on the current state of affairs or the current literature	F	30
Diagnostic Test Studies	Studies of the laboratory or filed performance of a diagnostic test. Generally compare sensitivity and specificity to some gold standard	G	0
Evidence Based guidelines	Guidelines developed by bodies after review of the literature	H	9
Expert Opinion, Editorials & Commentaries	Opinion or "newsy" narratives	I	27

**TOTAL**			**168**

### Elicitation of expert knowledge and opinion

While completing the literature review, we convened a meeting of experts on January 17 and 18, 2006, in Arlington, VA. Attendees represented a broad range of disciplines, including biomedical research, virology, clinical practice, infection control, epidemiology, public health, ethics, law, history, and health policy. All panelists excepting one were based in North America. Participants identified a set of non-pharmaceutical public health interventions that could potentially mitigate an influenza pandemic and grouped them into four categories: (1) infection control and prevention to reduce transmission when contact occurs between infected and uninfected individuals; (2) patient management to reduce contact between infected and uninfected individuals; (3) contact management to reduce contact between possibly infected and uninfected individuals; and (4) voluntary and mandatory community restrictions to reduce contact between groups that may contain infected persons (Table 3).

**Table 3:** Non-pharmaceutical public health interventions

**Human surveillance**
Case reporting
Early rapid viral diagnosis
Disinfection
Hand hygiene
Respiratory etiquette
Surgical and N95 Masks
Other personal protective equipment*
**Patient Management**
Isolation of sick individuals
Provision of social support services to the isolated
**Contact Management**
Quarantine†
Voluntary sheltering‡
Contact tracing
**Community Restrictions**
School closures
Workplace closures
Cancellation of group events
International and domestic travel restrictions

* Gowns, gloves and protective eye covers

† Separating exposed individuals from others

‡ Voluntary sequestration of healthy persons to avoid exposure

§Exit and entry screening, travel advisories

Participants qualitatively evaluated each intervention considering a broad range of factors, including efficacy (effects under ideal conditions) and various aspects of effectiveness (effects under real-world constraints). The latter included feasibility, cost, logistics, operational and infrastructure constraints, and acceptability in terms of concerns surrounding legality and ethics, equity, public confidence, and potential unintended consequences. In their deliberations, the experts also considered the different settings in which these interventions might be applied (healthcare facilities, community-based institutions, and private households). They also considered the epidemic phases in which the use of these interventions should be evaluated (no pandemic, no US pandemic, early localized US pandemic, and advanced US pandemic).

Following the meeting, we asked each expert to rate 200 unique intervention-setting-phase triads identified during the meeting based on the totality of their knowledge. Thirteen of seventeen responded. Ratings were on a scale of 1 to 5, with 1 being 'not recommended' and 5 being 'strongly recommended'. We scored the ratings using an adaptation of the RAND/UCLA Appropriateness Method [12,13]. We first discarded the two extreme high and low ratings for each item. We then defined agreement as all ratings falling within a single 2-point range and all other outcomes as disagreement. Among those items for which there was agreement, items with ratings of 4 or 5 were classified as a recommendation for use, and items with ratings of 1 or 2 were classified as a recommendation against use [12].

## Results

Our formal ratings of the articles revealed few high quality studies to inform the evidence base for non-pharmaceutical interventions for influenza. The majority of topically relevant articles we identified were narrative reviews, case reports, observational studies or expert opinion, editorials and commentaries (Table 2). We found only 9 systematic reviews of relevant material and 3 randomized clinical trials. Additionally, few of the topically relevant articles were directly on-point.

**Figure 1:**
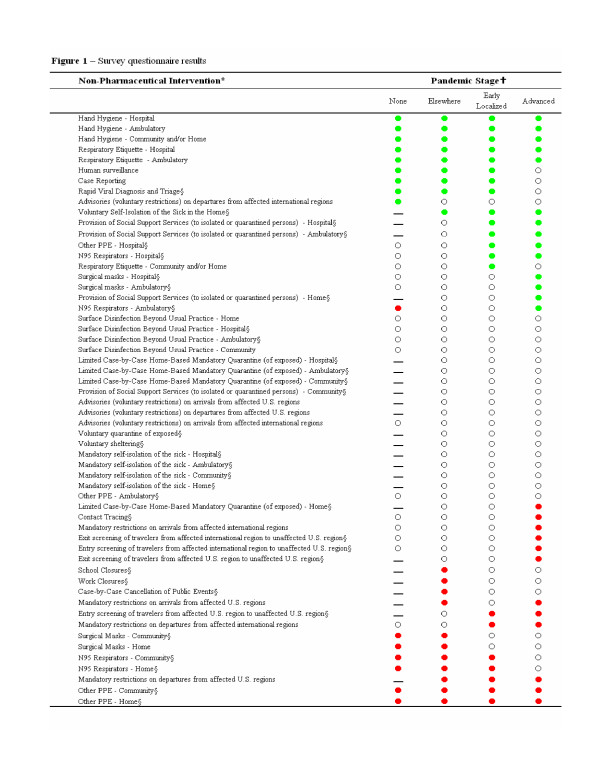
Survey questionnaire results. *Hospital = inpatient acute care hospital, inpatient long-term care facility or any inpatient setting; 'Ambulatory' = emergency departments, ambulatory hospital care, urgent care centers, providers' offices, clinics or other community-based healthcare settings and includes care delivered in the community by first responders; 'Community' = schools workplaces, churches, malls, stadiums, etc,; 'Home' = care delivered in private residences. †None = overseas cases only; Elsewhere = no cases in your state/locality/jurisdiction; Early Localized = cases your state/locality/jurisdiction; Advanced = widespread human-to-human transmission in the US §Since some items were left blank, the indicated results were based on 9, 10, 11 or 12 responses (of out of a possible 13). All remaining results are based on all 13 responses. Green Circle (insert '=') Recommendation for use (46/200 items (23%))Red Circle (insert '=') Recommendation against use (37/200 items (18.5%))(insert space) Clear Circle (insert '=')  Disagreement (117/200 items (59%) (insert space) Dash (insert '=') Not Applicable (respondents were instructed to leave blank)

In light of the evident lack of scientific evidence about specific non-pharmaceutical interventions in the context of seasonal or pandemic influenza, there was limited directly useable information from the majority of the studies identified in the formal Medline search. For this reason, we turned to expert opinion to inform and categorize the findings [14]. Expert panels are often used to develop guidelines and recommendations when compelling evidence is lacking. Drawing on both qualitative discussion at the expert panel and quantitative results from the follow-up survey discussed above, we classified the non-pharmaceutical interventions into two broad categories, those whose use was recommended by the panel and those whose use was not recommended. Table 4 (see Figure 1) summarizes the results from the survey questionnaire, providing the complete list of non-pharmaceutical intervention-setting-phase combinations that were queried and the number and proportion of items for which there was agreement (41.5%) or disagreement (58.5%). We included relevant findings from the literature, where available, in our discussion of the specific interventions, and we cited some of the selected studies from the formal Medline search, as well as others that supplemented the search, to provide necessary background information when appropriate and to support some commonly held views about infection control activities. We also note below interventions about which there was disagreement or no recommendation by the panel.

### Interventions recommended for use

#### Hand hygiene and respiratory etiquette

Hospital-based infection control measures such as hand hygiene and respiratory etiquette to prevent the spread of infection are widely supported in the literature and broadly accepted [15-20]. Many controlled studies have shown a protective effect of hand hygiene in reducing upper respiratory infections, although few of the infections studied were due to influenza [9,21-27]. Some studies suggest that use of an alcohol-based hand sanitizer is more effective in preventing carriage of non-spore forming bacteria and direct spread of most infections than antimicrobial soap or no hand washing, but antimicrobial handwashing products have not been shown to offer an advantage over soap and water [9,23,28-34].

The experts recommended rigorous and routine hand hygiene as an important strategy for healthcare workers and the general public in all settings and at all phases of a pandemic, including prior to a pandemic (Table 4 - see figure 1). However, important barriers to the effective use of hand hygiene were noted, including adherence, the supply and cost of commercially available disinfectant soaps and alcohol-based rubs, and the pervasive practice of hand-shaking. 

Experts also recommended respiratory etiquette as an important means of preventing transmission for all patients and providers in all pandemic phases, and in the community and/or home when the US pandemic is early and localized. Respiratory hygiene and cough etiquette is generally held to include covering the mouth and nose with a tissue or into the upper sleeve when coughing or sneezing, and refraining from spitting [35]. However, they urged that the promotion of respiratory etiquette be coupled with a compelling public education campaign that includes information regarding the signs and symptoms of influenza.

#### Human surveillance and case reporting

We found little direct empirical evidence on the efficacy or effectiveness of surveillance and case reporting in the context of influenza. Nevertheless, in light of experience with SARS, the experts recommended both as important to containing the spread of a pandemic [36]. Influenza surveillance supports a range of necessary preparedness activities, including: 1) providing information regarding the presence and epidemiology of influenza viruses in the community, 2) determining appropriate interventions, 3) targeting interventions, and 4) generating current accurate information for public health officials, providers and the public. While the experts agreed that human surveillance and case reporting are efficacious and likely to be effective during any pandemic phase, broad endorsement was qualified by concerns about resource constraints, especially in a large outbreak, potential difficulties in cooperation between providers and governmental and non-governmental entities, the cost of scaling up capacity to report and investigate influenza-like illness, privacy rights and the right to informed consent [5].

#### Rapid viral diagnosis

The key limitation of currently available rapid tests for influenza is suboptimal sensitivity, especially in adults [37-40]. New and more sensitive technologies for rapid diagnosis of influenza that can reliably identify influenza among patients with respiratory syndromes would greatly aid in the efficient allocation of limited resources such as isolation facilities. The experts agreed on the need for increased investment in the development and deployment of improved rapid diagnostic tests for influenza, arguing that such testing will be invaluable for effective surveillance and in managing all but the most advanced phases of a US pandemic. Moreover, since viral diagnosis of influenza is currently not routine practice, the experts reasoned that education regarding the importance of improved tests will be necessary to increase the adoption of such tests in the US health system. If new tests can be packaged in a way that facilitates use in non-clinical settings, their potential to facilitate disease containment efforts will be even greater. However, the lack of incentives for routine use of costly tests could limit development and production of new technologies, creating shortages in a pandemic emergency.

#### Provider and patient use of masks and other personal protective equipment

Uncertainty about the mode of influenza transmission has influenced debate about when and whether to use masks or N95 respirators for pandemic influenza. Droplet transmission is thought to be the primary mode of transmission, and provides the basis for CDC guidelines that health-care personnel wear masks for close patient contact (i.e., within 3 feet) to control influenza transmission during the influenza season [41]. But experience from seasonal influenza also provides evidence of contact, droplet and aerosol transmission of influenza that lend support for N95 respirators, which are designed to stop up to 95% of small airborne particles [42]. A recent Institute of Medicine (IOM) study found that empirical evidence about the efficacy or effectiveness of inexpensive, disposable masks and respirators against influenza is limited [43-46]. Our experts recognized this as an area of significant controversy and complexity, but they generally recommended reserving surgical masks, N95 respirators and other personal protective equipment for hospital and ambulatory patients and providers when a community outbreak begins or when the pandemic was widespread. Moreover, surgical masks and N95 respirators were recognized as a non-invasive technology that would induce no antiviral drug resistance. The experts qualified their recommendation, noting that poor training, improper use and, for N95 respirators, the need for fit-testing may compromise the overall effectiveness of these measures.

#### Isolation of the sick

The amount of influenza virus shed by symptomatic individuals is greater than in the asymptomatic phase, but viral shedding typically begins shortly after infection and before the onset of symptoms. This limits the efficacy of isolation except for individuals completely quarantined almost immediately after contact with an infected person [47]. However, more recent studies report that when numbers are small, isolation in hospitals using appropriate infection control measures may be effective[47]. While discussions were uniformly supportive of routine infection control measures, the experts did not agree on recommendations for *mandatory *isolation in any specific setting. This was because of the inconclusive nature of the evidence, the concern that healthcare facilities are likely to be rapidly overwhelmed, and that overflow into difficult to manage public settings such as arenas would be less effective. Moreover, mandatory isolation outside of healthcare settings, even if effective and enforceable, raises a range of legal, political and ethical issues that can, at a minimum, erode public acceptance of these policies. Despite the skepticism about mandatory isolation strategies, *voluntary *self-isolation in the home was recommended for all phases of a US pandemic.

### Interventions whose use is not recommended

#### Masks and other personal protective equipment for the general public

With the exception of some evidence from SARS, we did not find any published data that directly support the use of masks, respirators, or other personal protective equipment by the public, or other steps such as disinfecting surfaces beyond usual practices. The expert's views were mixed. There was uncertainty regarding requirements for masks or respirators because of uncertainty about the relative roles of droplet versus aerosol transmission. Concerns about supply, competency in mask and especially respirator fitting and use, adherence by the public, and social impact of mask-wearing all served to undermine the panel's confidence in the feasibility and acceptability of widespread use. On the survey, experts recommended against the use of masks or respirators by the public prior to the arrival of pandemic influenza and in the early localized phase. For similar reasons, experts recommended against the public use of other protective equipment such as gowns, gloves and protective eye wear.

#### Mandatory social distancing measures

Although social distancing measures such as workplace closures, limitations on location-based gatherings and events, and mandated travel restrictions have been a recent focus of investigation, and some of these measures were implemented in Asia and North America during SARS, their effectiveness in an influenza outbreak has not yet been established [5,8,48]. Despite the propensity of influenza epidemics to be amplified in primary schools, data on the effectiveness of school closures for reducing community transmission are contradictory. Most empirical studies suggest a decline in community transmission rates of respiratory infections with school closures[49,50], but the WHO Writing Group also noted older studies showing increases in the spread of disease and subsequent illness after a school holiday, and protective effects when schools remained open [9]. Recent modeling studies generally support school closure and confinement in the home as an effective means of reducing overall attack rates within communities when coupled with antiviral prophylaxis, but predicting the effect of closing schools and workplaces is difficult, since infectious individuals may be displaced into other settings[47,51,52]. Models suggest that cancellation of non-essential public gatherings and restrictions on long-distance travel might help to decrease rates of transmission and overall morbidity, but the effectiveness of these interventions has not been quantified.

The experts generally thought that community restrictions could be considered on a case-by-case basis, for example, cancellation of an event to which thousands would travel. However, efforts to forcibly limit public assembly or movement were seen as legally and ethically problematic, especially when there is limited scientific evidence supporting such restrictions. There are also important practical and logistical limitations to mandatory long-term community restrictions and compulsory quarantine, in addition to the problem of likely public opposition to such measures [5,53,54]. The experts recommended against these restrictions when outbreaks were elsewhere, and they did not agree otherwise. The same is true for school closures.

Consistent with the literature, the experts contended that widespread and sustained screening of travelers would ultimately be impractical and inefficient as long as detecting asymptomatic shedding is not feasible. Difficulties with the rapid diagnosis of influenza means that travel bans and screening programs risk detaining a large number of symptomatic persons who do not have influenza. There is also the possibility of such measures leading to an international backlash, decreasing cooperation at a time when increased is needed. However, voluntary measures and guidelines would likely be more acceptable and thus more effective. The experts recommended against any mandatory travel restrictions in the advanced phases of a pandemic and did not recommend restrictions on domestic or international departures or entry screening when a pandemic is in the early localized phase.

## Discussion

We evaluated the evidence-base for non-pharmaceutical public health interventions in an influenza pandemic by reviewing the recent scientific literature, convening a multidisciplinary meeting of experts, and eliciting expert knowledge qualitatively and quantitatively. Despite the poor quality of the evidence, the use of expert opinion has enabled us to identify strategies that are likely to help slow influenza transmission in a pandemic setting and also do no harm. Our findings highlight the importance of specifying the setting in which a non-pharmaceutical public health intervention will be used, as well as when its use should be considered. Although the interplay of these factors and deep uncertainty about the relative efficacy or effectiveness of specific non-pharmaceutical public health interventions prevents us from conclusively pinpointing an optimal set of non-pharmaceutical public health interventions for every circumstance, our study provides some important insights about pandemic planning.

Consistent with others, we found that the published literature revealed scant confirmatory evidence on efficacy and overall effectiveness of non-pharmaceutical public health interventions in an influenza pandemic, effectively forcing policymakers to turn to expert opinion[8,9]. Some infection control studies classified as systematic reviews, observational studies or evidence-based guidelines constituted stronger evidence, at least for spread of respiratory disease[14]. The remaining scientific evidence is of low strength (further research is very likely to change estimates of effect) or very low strength (effects are quite uncertain), specifically with respect to seasonal and pandemic influenza [14]. The published evidence consisted mainly of narrative reviews of past pandemics, contemporary observations, editorials, commentaries and case reports or series.  It also included articles drawing inferences from biology and pathophysiology, clinical epidemiology and mathematical modeling studies, rather than from randomized controlled trials evaluating interventions. In this context, expert opinion is particularly useful, and several clear messages emerged.

First, policymakers should actively promote personal responsibility for slowing spread of infection through good hand hygiene and respiratory etiquette in all settings and at all times. Use of disinfectant hand soaps and alcohol-based rubs should also be encouraged. Second, developing the capability and capacity for early rapid viral diagnosis should be a high priority. Third, healthcare providers need to be better trained to maximize the effectiveness of infection control measures, including use of masks, respirators, and other personal protective equipment. It may be reasonable to recommend limited use of personal protective measures in certain other settings such as mask-wearing by the ill or perhaps surface disinfection of very heavily trafficked public areas. However, other use of personal protective equipment by the general public is not recommended at this time. Fourth, and also consistent with other studies, we founds that widespread government mandates to segregate individuals, including isolation, quarantine, sheltering, location-based community restrictions, and travel restrictions, are less likely than voluntary measures to be recommended, especially over the longer-term[55]. Instead, less invasive voluntary efforts to reduce social contact, especially self-isolation of the sick at home, self-quarantine of the exposed, and, when feasible, sheltering by the well ought to be widely supported. This will require education, persuasion, and social support to ensure that medical and non-medical needs are met, with the latter being central to the success of sequestration measures in all settings. Fifth, information needs are pervasive. Very little of the literature is on point, and the experts disagreed 60 percent of the time. Well-controlled observational and especially interventional studies are needed, especially in the context of seasonal influenza.

## Conclusion

The demand for scientific evidence on non-pharmaceutical public health interventions for influenza is pervasive, and policy recommendations must rely heavily on expert judgment. In the absence of a definitive science base, our assessment of the evidence identified areas for further investigation as well as non-pharmaceutical public health interventions that experts believe are likely to be beneficial, feasible, and socially and politically acceptable in an influenza pandemic. Taken together, the literature and expert opinion reveal the kinds of explicit judgments required to translate existing knowledge into policy-relevant terms. These findings should be considered in forming national, state, local, and facility pandemic plans.

## Competing interests

The author(s) declare that they have no competing interests.

## Authors' contributions

JEA designed and carried out the literature review, designed and conducted the expert panel and drafted the manuscript. NL conceived of the study, and participated in its design and coordination, conducted the expert panel, and helped to draft the manuscript. JW conceived of the study, and participated in its design and coordination, and helped to draft the manuscript. SAB designed the literature review, designed and conducted the expert panel, designed the follow-up expert survey and analysis plan, and helped to draft the manuscript. All authors read and approved the final manuscript.

## Pre-publication history

The pre-publication history for this paper can be accessed here:



## Supplementary Material

Additional file 1 Search terms. The file contains a brief paragraph of the Medline search terms. Click here for file

Additional file 2 Complete list of final articles reviewed and classified (N = 168). This file contains a complete list of the final set of articles from our search that were reviewed and classified. Click here for file
