# Supplementary material for: Non-pharmaceutical public health interventions for pandemic influenza: an evaluation of the evidence base
Source: BMC Public Health. 2007 Aug 15;7:208. doi: 10.1186/1471-2458-7-208 (PMC2040158; doi:10.1186/1471-2458-7-208)
Supplement: Additional file 1 — Search terms. The file contains a brief paragraph of the Medline search terms. [file 1471-2458-7-208-S1.pdf]

Additional file 1 – Search terms

We searched various combinations of the following Medical Subject Headings (MeSH) disease and intervention descriptors back to 1966: "Common cold", "influenza", "pandemic", "viral pneumonia", "severe acute respiratory syndrome", "SARS", "laryngeal tuberculosis", "pulmonary tuberculosis", "respiratory tract infection", "bronchitis", "bronchiolities", "legionnaires disease", "pharyngitis", "pleurisy", "tracheitis", "whooping cough", "orthomyxoviridae", "communicable disease", "infectious disease", "disease transmission", "contact tracing", "disease notification", "infection control", "handwashing", "patient isolation", "quarantine", "border", "travel", "dental infection control", "dental", "sterilization", "sanitation", "universal precautions", "mandatory programs", "protective devices", "masks", "protective clothing", "gloves", "personal protective equipment", "respiratory protective devices", "prevention and control", "hygiene" , "school closure", "transmission", "disease outbreak", "entry screen."
