# Supplementary material for: Non-pharmaceutical public health interventions for pandemic influenza: an evaluation of the evidence base
Source: BMC Public Health. 2007 Aug 15;7:208. doi: 10.1186/1471-2458-7-208 (PMC2040158; doi:10.1186/1471-2458-7-208)
Supplement: Additional file 2 — Complete list of final articles reviewed and classified (N = 168). This file contains a complete list of the final set of articles from our search that were reviewed and classified. [file 1471-2458-7-208-S2.pdf]

Additional file 2 – Complete list of final articles reviewed and classified (N = 168)

(1997). "Draft guideline for infection control in health care personnel, 1997--CDC. Notice." Fed Regist **62**(173): 47276-327.

(2001). "From the Centers for Disease Control and Prevention. Influenza activity--United States, 2000-01 season." Jama **285**(14): 1832-3.

(2003). "Communicable disease and health protection quarterly review: July-September 2002. From the PHLS Communicable Disease Surveillance Centre." J Public Health Med **25**(1): 76-82.

(2003). "Emerging stronger from the China crisis." Lancet **361**(9366): 1311.

(2003). "From the Centers for Disease Control and Prevention. Severe Acute Respiratory Syndrome (SARS) and coronavirus testing--United States, 2003." Jama **289**(17): 2203-6.

(2003). "From the Centers for Disease Control and Prevention. Update: outbreak of severe acute respiratory syndrome--worldwide, 2003." Jama **289**(15): 1918-20.

(2003). "From the Centers for Disease Control and Prevention. Update: outbreak of severe acute respiratory syndrome--worldwide, 2003." JAMA **289**(16): 2059-60.

(2003). "From the Centers for Disease Control and Prevention. Update: severe acute respiratory syndrome--United States, 2003." Jama **289**(18): 2350-1.

(2003). "From the Centers for Disease Control and Prevention. Update: severe acute respiratory syndrome--United States, 2003." JAMA **289**(19): 2495, 2498.

(2003). "From the Centers for Disease Control and Prevention. Update: severe acute respiratory syndrome--United States, May 14, 2003." Jama **289**(21): 2790.

(2003). "From the Centers for Disease Control and Prevention. Update: Severe acute respiratory syndrome--United States, May 21, 2003." Jama **289**(22): 2932.

(2003). "From the Centers for Disease Control and Prevention. Updated interim surveillance case definition for severe acute respiratory syndrome (SARS)--United States, April 29, 2003." Jama **289**(20): 2637-9.

(2003). "A multicentre collaboration to investigate the cause of severe acute respiratory syndrome." Lancet **361**(9370): 1730-3.

(2003). "A new vision for human security." Lancet **361**(9370): 1665.

(2003). "Will SARS hurt the world's poor?" Lancet **361**(9368): 1485.

(2004). "Emerging infectious diseases including severe acute respiratory syndrome

(SARS): guidelines for commercial air travel and air medical transport." Aviat Space Environ Med **75**(1): 85-6.

(2005). "Strategies adopted and lessons learnt during the severe acute respiratory syndrome crisis in Singapore." Rev Med Virol **15**(1): 57-70.

Abdullah, A. S., B. Tomlinson, et al. (2003). "Lessons from the severe acute respiratory syndrome outbreak in Hong Kong." Emerg Infect Dis **9**(9): 1042-5.

Asaolu, S. O. and I. E. Ofoezie (2003). "The role of health education and sanitation in the control of helminth infections." Acta Trop **86**(2-3): 283-94.

Barnitz, L. and M. Berkwits (2006). "The health care response to pandemic influenza." Ann Intern Med **145**(2): 135-7.

Barrett, C. L., S. G. Eubank, et al. (2005). "If smallpox strikes Portland." Sci Am **292**(3): 42-9.

Beck, M. A., J. Handy, et al. (2004). "Host nutritional status: the neglected virulence factor." Trends Microbiol **12**(9): 417-23.

Beckett, W. S. (2000). "Occupational respiratory diseases." N Engl J Med **342**(6): 406-13.

Beigel, J. H., J. Farrar, et al. (2005). "Avian influenza A (H5N1) infection in humans." N Engl J Med **353**(13): 1374-85.

Benitez, M. A. (2003). "Beijing doctor alleges SARS cases cover-up in China." Lancet **361**(9366): 1357.

Benitez, M. A. (2003). "Hong Kong bears brunt of latest outbreak." Lancet **361**(9362): 1018.

Bettin, K., C. Clabots, et al. (1994). "Effectiveness of liquid soap vs. chlorhexidine gluconate for the removal of Clostridium difficile from bare hands and gloved hands." Infect Control Hosp Epidemiol **15**(11): 697-702.

Blendon, R. J., J. M. Benson, et al. (2004). "The public's response to severe acute respiratory syndrome in Toronto and the United States." Clin Infect Dis **38**(7): 925-31.

Bloomfield, S. F. (2001). "Preventing infectious disease in the domestic setting: a risk-based approach." Am J Infect Control **29**(4): 207-10.

Boyce, J. M. and D. Pittet (2002). "Guideline for Hand Hygiene in Health-Care Settings. Recommendations of the Healthcare Infection Control Practices Advisory Committee and the HICPAC/SHEA/APIC/IDSA Hand Hygiene Task Force. Society for Healthcare Epidemiology of America/Association for Professionals in

Infection Control/Infectious Diseases Society of America." MMWR Recomm Rep **51**(RR-16): 1-45, quiz CE1-4.

Brownstein, J. S., C. J. Wolfe, et al. (2006). "Empirical evidence for the effect of airline travel on inter-regional influenza spread in the United States." PLoS Med **3**(10): e401.

Brundage, J. F. (2006). "Interactions between influenza and bacterial respiratory pathogens: implications for pandemic preparedness." Lancet Infect Dis **6**(5): 303-12.

Buckley, H. (2003). "Clinical brief: severe acute respiratory syndrome (SARS)." Aaohn J **51**(7): 303-5.

Butler, D. (2005). "Drugs could head off a flu pandemic--but only if we respond fast enough." Nature **436**(7051): 614-5.

Butler, J. C., P. H. Kilmarx, et al. (1996). "Perspectives in fatal epidemics." Infect Dis Clin North Am **10**(4): 917-37.

Camitz, M. and F. Liljeros (2006). "The effect of travel restrictions on the spread of a moderately contagious disease." BMC Med **4**: 32.

Carrat, F., J. Luong, et al. (2006). "A 'small-world-like' model for comparing interventions aimed at preventing and controlling influenza pandemics." BMC Med **4**: 26.

Castillo-Chavez, C., C. W. Castillo-Garsow, et al. (2003). "MSJAMA. Mathematical models of isolation and quarantine." Jama **290**(21): 2876-7.

Chan-Yeung, M. (2004). "Severe acute respiratory syndrome (SARS) and healthcare workers." Int J Occup Environ Health **10**(4): 421-7.

Chan-Yeung, M., G. C. Ooi, et al. (2003). "Severe acute respiratory syndrome." Int J Tuberc Lung Dis **7**(12): 1117-30.

Chan-Yeung, M. and R. H. Xu (2003). "SARS: epidemiology." Respirology **8 Suppl**: S9-14.

Chen, S. Y., C. P. Su, et al. (2004). "Predictive model of diagnosing probable cases of severe acute respiratory syndrome in febrile patients with exposure risk." Ann Emerg Med **43**(1): 1-5.

Cole, E. C. and C. E. Cook (1998). "Characterization of infectious aerosols in health care facilities: an aid to effective engineering controls and preventive strategies." Am J Infect Control **26**(4): 453-64.

Collins, B. J. (1988). "The hospital environment: how clean should a hospital be?" The

Journal of hospital infection **11 Suppl A**: 53-6.

Corwin, A., C. H. Simanjuntak, et al. (1997). "Emerging disease surveillance in Southeast Asia." Ann Acad Med Singapore **26**(5): 628-31.

Couch, R. B. (1984). "The common cold: control?" J Infect Dis **150**(2): 167-73.

Couch, R. B., T. R. Cate, et al. (1966). "Effect of route of inoculation on experimental respiratory viral disease in volunteers and evidence for airborne transmission." Bacteriological reviews **30**(3): 517-29.

Day, T., A. Park, et al. (2006). "When is quarantine a useful control strategy for emerging infectious diseases?" Am J Epidemiol **163**(5): 479-85.

DeHart, R. L. (2003). "Health issues of air travel." Annu Rev Public Health **24**: 133-51.

Diamond, B. (2003). "SARS spreads new outlook on quarantine models." Nat Med **9**(12): 1441.

Doebbeling, B. N., M. A. Pfaller, et al. (1988). "Removal of nosocomial pathogens from the contaminated glove. Implications for glove reuse and handwashing." Ann Intern Med **109**(5): 394-8.

Doebbeling, B. N., G. L. Stanley, et al. (1992). "Comparative efficacy of alternative hand-washing agents in reducing nosocomial infections in intensive care units." N Engl J Med **327**(2): 88-93.

Donnelly, C. A., A. C. Ghani, et al. (2003). "Epidemiological determinants of spread of causal agent of severe acute respiratory syndrome in Hong Kong." Lancet **361**(9371): 1761-6.

Drinka, P. J., P. Krause, et al. (1996). "Report of an outbreak: nursing home architecture and influenza-A attack rates." J Am Geriatr Soc **44**(8): 910-3.

Dye, C. and N. Gay (2003). "Epidemiology. Modeling the SARS epidemic." Science **300**(5627): 1884-5.

Enserink, M. (2003). "Breakthrough of the year. SARS: a pandemic prevented." Science **302**(5653): 2045.

Enserink, M. (2003). "Infectious diseases. WHO wants 21st-century reporting regs." Science **300**(5620): 717-8.

Enserink, M. (2005). "Epidemiology. Drugs, quarantine might stop a pandemic before it starts." Science **309**(5736): 870-1.

Ferguson, N. M., D. A. Cummings, et al. (2005). "Strategies for containing an emerging influenza pandemic in Southeast Asia." Nature **437**(7056): 209-14.

Ferson, M. J. (1997). "Infection control in child care settings." Commun Dis Intell **21**(22): 333-7.

Flahault, A., E. Vergu, et al. (2006). "Strategies for containing a global influenza pandemic." Vaccine **24**(44-46): 6751-5.

Fleming, D. (2005). "Influenza pandemics and avian flu." Bmj **331**(7524): 1066-9.

Freedman, D. O. and J. Woodall (1999). "Emerging infectious diseases and risk to the traveler." Med Clin North Am **83**(4): 865-83, v.

Friedman, C., M. Barnette, et al. (1999). "Requirements for infrastructure and essential activities of infection control and epidemiology in out-of-hospital settings: a consensus panel report. Association for Professionals in Infection Control and Epidemiology and Society for Healthcare Epidemiology of America." Infect Control Hosp Epidemiol **20**(10): 695-705.

Gallaher, S. (2005). "SARS: What We Have Learned So Far." Dimens Crit Care Nurs **24**(2): 51-4; quiz 55-6.

Galvani, A. P. (2004). "Emerging infections: what have we learned from SARS?" Emerg Infect Dis **10**(7): 1351-2.

Garner, J. S. (1996). "Guideline for isolation precautions in hospitals. The Hospital Infection Control Practices Advisory Committee." Infect Control Hosp Epidemiol **17**(1): 53-80.

Gensheimer, K. F., M. I. Meltzer, et al. (2003). "Influenza pandemic preparedness." Emerg Infect Dis **9**(12): 1645-8.

Goldmann, D. A. (2001). "Epidemiology and prevention of pediatric viral respiratory infections in health-care institutions." Emerg Infect Dis **7**(2): 249-53.

Goldmann, D. A., R. A. Weinstein, et al. (1996). "Strategies to Prevent and Control the Emergence and Spread of Antimicrobial-Resistant Microorganisms in Hospitals. A challenge to hospital leadership." Jama **275**(3): 234-40.

Goodman, R. A. and S. L. Solomon (1991). "Transmission of infectious diseases in outpatient health care settings." Jama **265**(18): 2377-81.

Gopalakrishna, G., P. Choo, et al. (2004). "SARS transmission and hospital containment." Emerg Infect Dis **10**(3): 395-400.

Gostin, L. (2006). "Public health strategies for pandemic influenza: ethics and the law." Jama **295**(14): 1700-4.

Gostin, L. O. (2006). "Medical countermeasures for pandemic influenza: ethics and the law." Jama **295**(5): 554-6.

Gostin, L. O., R. Bayer, et al. (2003). "Ethical and legal challenges posed by severe acute respiratory syndrome: implications for the control of severe infectious disease threats." Jama **290**(24): 3229-37.

Gray, G. C., J. D. Callahan, et al. (1999). "Respiratory diseases among U.S. military personnel: countering emerging threats." Emerg Infect Dis **5**(3): 379-85.

Greaves, F. (2004). "What are the most appropriate methods of surveillance for monitoring an emerging respiratory infection such as SARS?" J Public Health (Oxf) **26**(3): 288-92.

Gubler, D. J. (1998). "Dengue and dengue hemorrhagic fever." Clin Microbiol Rev **11**(3): 480-96.

Gupta, A. G., C. A. Moyer, et al. (2005). "The economic impact of quarantine: SARS in Toronto as a case study." J Infect **50**(5): 386-93.

Habib, N. A. and R. H. Behrens (2000). "Respiratory infections in the traveler." Curr Opin Pulm Med **6**(3): 246-9.

Hawryluck, L., W. L. Gold, et al. (2004). "SARS control and psychological effects of quarantine, Toronto, Canada." Emerg Infect Dis **10**(7): 1206-12.

Hendley, J. O. and J. M. Gwaltney, Jr. (1988). "Mechanisms of transmission of rhinovirus infections." Epidemiol Rev **10**: 243-58.

Herwaldt, L. A., S. D. Smith, et al. (1998). "Infection control in the outpatient setting." Infect Control Hosp Epidemiol **19**(1): 41-74.

Hsueh, P. R. and P. C. Yang (2005). "Severe acute respiratory syndrome epidemic in Taiwan, 2003." J Microbiol Immunol Infect **38**(2): 82-8.

Hui, D. S., M. Ip, et al. (2006). "Airflows around oxygen masks: A potential source of infection?" Chest **130**(3): 822-6.

Inouye, S., Y. Matsudaira, et al. (2006). "Masks for influenza patients: measurement of airflow from the mouth." Jpn J Infect Dis **59**(3): 179-81.

Jeffries, D. J. (1995). "Viral hazards to and from health care workers." J Hosp Infect **30 Suppl**: 140-55.

Keeler, N. and J. Lingappa (2004). "Severe acute respiratory syndrome: public health response and clinical practice update for an emerging disease." Curr Opin Pediatr **16**(1): 61-9.

Kjolen, H. and B. M. Andersen (1992). "Handwashing and disinfection of heavily contaminated hands--effective or ineffective?" J Hosp Infect **21**(1): 61-71.

Kondro, W. (2003). "Canada fury at WHO ban." Lancet **361**(9368): 1525.

Kondro, W. (2003). "Canadians still stung by WHO's SARS travel advisory." Lancet **361**(9369): 1624.

Lacey, C. (2003). "Abuse of quarantine authority. The case for a federal approach to infectious disease containment." J Leg Med **24**(2): 199-214.

Lapinsky, S. E. and J. T. Granton (2004). "Critical care lessons from severe acute respiratory syndrome." Curr Opin Crit Care **10**(1): 53-8.

Larson, E. (1989). "Handwashing: it's essential--even when you use gloves." Am J Nurs **89**(7): 934-9.

Larson, E. and E. K. Kretzer (1995). "Compliance with handwashing and barrier precautions." J Hosp Infect **30 Suppl**: 88-106.

Larson, E. L., J. L. Bryan, et al. (1997). "A multifaceted approach to changing handwashing behavior." Am J Infect Control **25**(1): 3-10.

Larson, E. L., P. I. Eke, et al. (1986). "Efficacy of alcohol-based hand rinses under frequent-use conditions." Antimicrob Agents Chemother **30**(4): 542-4.

Leder, K. and D. Newman (2005). "Respiratory infections during air travel." Intern Med J **35**(1): 50-5.

Leder, K., V. Sundararajan, et al. (2003). "Respiratory tract infections in travelers: a review of the GeoSentinel surveillance network." Clin Infect Dis **36**(4): 399-406.

Letts, J. (2006). "Ethical challenges in planning for an influenza pandemic." N S W Public Health Bull **17**(9-10): 131-4.

Levi, J. and T. Inglesby (2006). "Working Group on Pandemic Influenza Preparedness: joint statement in response to Department of Health and Human Services Pandemic Influenza Plan." Clin Infect Dis **42**(1): 92-4.

Li, Y., G. M. Leung, et al. (2007). "Role of ventilation in airborne transmission of infectious agents in the built environment - a multidisciplinary systematic review." Indoor Air **17**(1): 2-18.

Liang, W., Z. Zhu, et al. (2004). "Severe acute respiratory syndrome, Beijing, 2003." Emerg Infect Dis **10**(1): 25-31.

Lipsitch, M., T. Cohen, et al. (2003). "Transmission dynamics and control of severe acute respiratory syndrome." Science **300**(5627): 1966-70.

Lo, B. and M. H. Katz (2005). "Clinical decision making during public health emergencies: ethical considerations." Ann Intern Med **143**(7): 493-8.

Longini, I. M., Jr., A. Nizam, et al. (2005). "Containing pandemic influenza at the source." Science **309**(5737): 1083-7.

Loutfy, M. R., T. Wallington, et al. (2004). "Hospital preparedness and SARS." Emerg Infect Dis **10**(5): 771-6.

Luna, L. K., M. Panning, et al. (2007). "Spectrum of viruses and atypical bacteria in intercontinental air travelers with symptoms of acute respiratory infection." J Infect Dis **195**(5): 675-9.

Malone, N. and E. Larson (1996). "Factors associated with a significant reduction in hospital-wide infection rates." Am J Infect Control **24**(3): 180-5.

Mangili, A. and M. A. Gendreau (2005). "Transmission of infectious diseases during commercial air travel." Lancet **365**(9463): 989-96.

Markel, H., A. M. Stern, et al. (2006). "Nonpharmaceutical influenza mitigation strategies, US communities, 1918-1920 pandemic." Emerg Infect Dis **12**(12): 1961-4.

McGeer, A. (2004). "Let him who desires peace prepare for war: United States hospitals and severe acute respiratory syndrome preparedness." Clin Infect Dis **39**(2): 275-7.

McInnes, K. and L. Safian (2005). "Keeping SARS out: an education program for SARS screeners in one Ontario hospital." J Nurses Staff Dev **21**(2): 73-8.

McNeil, S. A., C. L. Foster, et al. (2001). "Effect of hand cleansing with antimicrobial soap or alcohol-based gel on microbial colonization of artificial fingernails worn by health care workers." Clin Infect Dis **32**(3): 367-72.

Meibalane, R., G. V. Sedmak, et al. (1977). "Outbreak of influenza in a neonatal intensive care unit." J Pediatr **91**(6): 974-6.

Miller, J. M., T. W. Tam, et al. (2000). "Cruise ships: high-risk passengers and the global spread of new influenza viruses." Clin Infect Dis **31**(2): 433-8.

Misrahi, J. J., J. A. Foster, et al. (2004). "HHS/CDC legal response to SARS outbreak." Emerg Infect Dis **10**(2): 353-5.

Mitka, M. (2003). "SARS thrusts quarantine into the limelight." Jama **290**(13): 1696-8.

Moore, D., B. Gamage, et al. (2005). "Protecting health care workers from SARS and other respiratory pathogens: organizational and individual factors that affect adherence to infection control guidelines." Am J Infect Control **33**(2): 88-96.

Morse, L. J. and L. E. Schonbeck (1968). "Hand lotions--a potential nosocomial hazad." N Engl J Med **278**(7): 376-8.

Morse, S. S. (1995). "Factors in the emergence of infectious diseases." Emerg Infect Dis **1**(1): 7-15.

Morse, S. S., R. L. Garwin, et al. (2006). "Public health. Next flu pandemic: what to do until the vaccine arrives?" Science **314**(5801): 929.

Musher, D. M. (2003). "How contagious are common respiratory tract infections?" N Engl J Med **348**(13): 1256-66.

Mylotte, J. M. (2002). "Nursing home-acquired pneumonia." Clin Infect Dis **35**(10): 1205-11.

Oh, V. M. and T. K. Lim (2003). "Singapore's experience of SARS." Clin Med **3**(5): 448-51.

Okeke, I. N., K. P. Klugman, et al. (2005). "Antimicrobial resistance in developing countries. Part II: strategies for containment." Lancet Infect Dis **5**(9): 568-80.

Olsen, R. J., P. Lynch, et al. (1993). "Examination gloves as barriers to hand contamination in clinical practice." Jama **270**(3): 350-3.

Olsen, S. J., H. L. Chang, et al. (2003). "Transmission of the severe acute respiratory syndrome on aircraft." N Engl J Med **349**(25): 2416-22.

Pang, X., Z. Zhu, et al. (2003). "Evaluation of control measures implemented in the severe acute respiratory syndrome outbreak in Beijing, 2003." Jama **290**(24): 3215-21.

Peiris, J. S. M., K. Y. Yuen, et al. (2003). "The severe acute respiratory syndrome." The New England journal of medicine **349**(25): 2431-41.

Pourbohloul, B., L. A. Meyers, et al. (2005). "Modeling control strategies of respiratory pathogens." Emerg Infect Dis **11**(8): 1249-56.

Rabie, T. and V. Curtis (2006). "Handwashing and risk of respiratory infections: a quantitative systematic review." Trop Med Int Health **11**(3): 258-67.

Rhinehart, E. (2001). "Infection control in home care." Emerg Infect Dis **7**(2): 208-11.

Roselle, G. A., L. H. Danko, et al. (2000). "Tuberculosis in the veterans healthcare system: a six-year review and evaluation of programme effectiveness." Epidemiol Infect **125**(2): 315-23.

Royal, L. and I. McCoubrey (1989). "International spread of disease by air travel." Am Fam Physician **40**(5): 129-36.

Sepkowitz, K. A. (1996). "Occupationally acquired infections in health care workers. Part I." Ann Intern Med **125**(10): 826-34.

Sepkowitz, K. A. (1996). "Occupationally acquired infections in health care workers. Part II." Ann Intern Med **125**(11): 917-28.

Seto, W. H., D. Tsang, et al. (2003). "Effectiveness of precautions against droplets and contact in prevention of nosocomial transmission of severe acute respiratory syndrome (SARS)." Lancet **361**(9368): 1519-20.

Shortridge, K. F. (1995). "The next pandemic influenza virus?" Lancet **346**(8984): 1210-2.

Simmons, B., M. Trusler, et al. (1990). "Infection control for home health." Infect Control Hosp Epidemiol **11**(7): 362-70.

Srinivasan, A., D. B. Jernign, et al. (2004). "Hospital preparedness for severe acute respiratory syndrome in the United States: views from a national survey of infectious diseases consultants." Clin Infect Dis **39**(2): 272-4.

Srinivasan, A., L. C. McDonald, et al. (2004). "Foundations of the severe acute respiratory syndrome preparedness and response plan for healthcare facilities." Infect Control Hosp Epidemiol **25**(12): 1020-5.

Stiver, H. G. (2004). "The threat and prospects for control of an influenza pandemic." Expert Rev Vaccines **3**(1): 35-42.

Svoboda, T., B. Henry, et al. (2004). "Public health measures to control the spread of the severe acute respiratory syndrome during the outbreak in Toronto." N Engl J Med **350**(23): 2352-61.

Tan, X., S. Li, et al. (2004). "Severe acute respiratory syndrome epidemic and change of people's health behavior in China." Health Educ Res **19**(5): 576-80.

Tang, J. W., Y. Li, et al. (2006). "Factors involved in the aerosol transmission of infection and control of ventilation in healthcare premises." J Hosp Infect **64**(2): 100-14.

Tellier, R. (2006). "Review of aerosol transmission of influenza A virus." Emerg Infect Dis **12**(11): 1657-62.

Top, F. H., Jr. and P. K. Russell (1977). "Swine influenza A at Fort Dix, New Jersey (January-February 1976). IV. Summary and speculation." J Infect Dis **136 Suppl**: S376-80.

Trampuz, A., R. M. Prabhu, et al. (2004). "Avian influenza: a new pandemic threat?" Mayo Clin Proc **79**(4): 523-30; quiz 530.

Twu, S. J., T. J. Chen, et al. (2003). "Control measures for severe acute respiratory syndrome (SARS) in Taiwan." Emerg Infect Dis **9**(6): 718-20.

Valenti, W. M. and M. A. Menegus (1981). "Nosocomial viral infections: IV. Guidelines for cohort isolation, the communicable disease survey, collection, and transport of specimens for virus isolation, and considerations for the future." Infect Control **2**(3): 236-45.

Vogel, G. (2003). "SARS outbreak. Modelers struggle to grasp epidemic's potential scope." Science **300**(5619): 558-9.

Wade, J. J., N. Desai, et al. (1991). "Hygienic hand disinfection for the removal of epidemic vancomycin-resistant Enterococcus faecium and gentamicin-resistant Enterobacter cloacae." J Hosp Infect **18**(3): 211-8.

Watts, J. (2003). "China takes drastic action over SARS threat." Lancet **361**(9370): 1708-9.

Webby, R. J. and R. G. Webster (2003). "Are we ready for pandemic influenza?" Science **302**(5650): 1519-22.

Weingarten, S., M. Friedlander, et al. (1988). "Influenza surveillance in an acute-care hospital." Arch Intern Med **148**(1): 113-6.

Weinstein, R. A. (2004). "Planning for epidemics--the lessons of SARS." N Engl J Med **350**(23): 2332-4.

Wenzel, R. P. and M. B. Edmond (2003). "Managing SARS amidst uncertainty." N Engl J Med **348**(20): 1947-8.

Williams, J. R., P. Y. Chen, et al. (2002). "Influenza: prospect for prevention and control." Kaohsiung J Med Sci **18**(9): 421-34.

World Health Organization, W. G. (2006). "Nonpharmaceutical Interventions for Pandemic Flu." Emerging Infectious Diseases **12**(1): 81-87.

World Health Organization, W. G. (2006). "Pandemic Influenza, National and Community Measures." Emerging Infectious Diseases **12**(1): 88-94.

Wright, S. A. and V. M. Bieluch (1993). "Selected nosocomial viral infections." Heart Lung **22**(2): 183-7.

Wu, J. T., S. Riley, et al. (2006). "Reducing the impact of the next influenza pandemic using household-based public health interventions." PLoS Med **3**(9): e361.

Yoshikawa, T. T. and D. C. Norman (1995). "Infection control in long-term care." Clin Geriatr Med **11**(3): 467-80.

Yu, I. T. and J. J. Sung (2004). "The epidemiology of the outbreak of severe acute respiratory syndrome (SARS) in Hong Kong--what we do know and what we don't." Epidemiol Infect **132**(5): 781-6.

Zambon, M. and K. G. Nicholson (2003). "Sudden acute respiratory syndrome." Bmj **326**(7391): 669-70.

Zhong, N. S. and G. W. Wong (2004). "Epidemiology of severe acute respiratory syndrome (SARS): adults and children." Paediatr Respir Rev **5**(4): 270-4.

Zitter, J. N., P. D. Mazonson, et al. (2002). "Aircraft cabin air recirculation and symptoms of the common cold." Jama **288**(4): 483-6.
